# Longitudinal network structure of depression symptoms and self-efficacy in low-income mothers

**DOI:** 10.1371/journal.pone.0191675

**Published:** 2018-01-23

**Authors:** Hudson P. Santos, Jolanda J. Kossakowski, Todd A. Schwartz, Linda Beeber, Eiko I. Fried

**Affiliations:** 1 School of Nursing, University of North Carolina, Chapel Hill, North Carolina, United States of America; 2 Department of Psychological Methods, University of Amsterdam, Amsterdam, The Netherlands; 3 Department of Biostatistics, Gillings School of Global Public Health, University of North Carolina, Chapel Hill, North Carolina, United States of America; Istituto Superiore Di Sanita, ITALY

## Abstract

Maternal depression was recently conceptualized as a network of interacting symptoms. Prior studies have shown that low self-efficacy, as an index of maternal functioning, is one important source of stress that worsens depression. We have limited information, however, on the specific relationships between depression symptoms and self-efficacy. In this study, we used regularized partial correlation networks to explore the multivariate relationships between maternal depression symptoms and self-efficacy over time. Depressed mothers (n = 306) completed the Center for Epidemiological Studies Depression (CES-D) scale at four time points, between four and eight weeks apart. We estimated (a) the network structure of the 20 CES-D depression symptoms and self-efficacy for each time point, (b) determined the centrality or structural importance of all variables, and (c) tested whether the network structure changed over time. In the resulting networks, self-efficacy was mostly negatively connected with depression symptoms. The strongest relationships among depression symptoms were ‘lonely—sleep difficulties’ and ‘inability to get going—crying’. ‘Feeling disliked’ and ‘concentration difficulty’ were the two most central symptoms. In comparing the network structures, we found that the network structures were moderately stable over time. This is the first study to investigate the network structure and their temporal stability of maternal depression symptoms and self-efficacy in low-income depressed mothers. We discuss how these findings might help future research to identify clinically relevant symptom-to-symptom relationships that could drive maternal depression processes, and potentially inform tailored interventions. We share data and analytical code, making our results fully reproducible.

## Introduction

Motherhood is often associated with cultural expectations of happiness and satisfaction. These expectations are at odds with the evidence that early motherhood is associated with elevated levels of maternal depression symptoms after childbirth. In fact, maternal depression is the most common mental health complication for mothers worldwide, with prevalence rates at 10–15% in high income countries [1], and higher rates reported in low-income countries (e.g., Chile: 37.4%; South Africa: 36.5%; India: 32.4%; [2]). Maternal depression is associated with substantial morbidity for mother, infant, and family that includes increased risk for impaired parenting behavior, infant development, and can lead to suicide and/or infanticide [3, 4].

In addition to depression symptoms, low self-efficacy can affect the functional status of mothers [5]. Self-efficacy, which refers to the belief individuals have in their abilities to successfully perform their duties [6, 7], such as parenting, has been found to be inversely related with maternal depression [8, 9]. Self-efficacy is an important index for the successful transition to motherhood and is an important factor related to positive maternal behavior [10–12]. The cumulative effects of low self-efficacy over time are believed to contribute to persistent depression symptoms [10, 13]. However, no evidence is available on the relationship of self-efficacy and specific depression symptoms, since research to date has investigated the association between self-efficacy and depression severity *sum-scores*. The goal of this paper is thus to explore the specific interactions between self-efficacy and individual maternal depression symptoms, and test whether these are consistent over time.

Statistically, maternal depression has been largely modeled by way of reflective measurement models [14, 15], where one underlying latent variable gives rise to the correlations among symptoms. This is based on the conceptualization that psychiatric illnesses such as depression are common causes of their respective symptoms, and this has been the main driver of research efforts to discover the underlying mechanism and bio-signatures of depression [16]. In line with this conceptualization, clinical research frequently represents depression severity as the sum-scores of symptoms assessed using self-reports or observer rating scales, while the diagnosis of depression is represented as a dichotomous (yes or no) categorization based on clinical interviews. Either way, the variations of symptom patterns are lost, and so is the ability to investigate whether specific symptoms differ in various domains [17].

Perhaps one of the most important insight gained from recent depression research is that depression can be understood as a complex system [18], due to the complex relationships among individual symptoms [16]. In other words, the quality and configuration of individual depression symptoms are important factors because symptoms can lead to differential impairment in function, have differential underlying biology, and play differential roles in the longitudinal course of depression episodes [19–21]. Embracing the complex nature of multivariate relationships of depression symptomatology, symptom network models (symptoms—set of nodes, connected by pairwise associations—set of edges) as a framework in which depression *is* the interaction among its symptoms are being developed [22, 23], along with a newly proposed network theory of mental disorders by Borsboom [22]. The resulting network of complex symptom interactions can not only inform how specific symptoms are interacting among themselves [17, 22, 24], but also how symptoms relate to “external” variables, such as self-eficacy, grief, and hormonal markers [22, 25–27]. The relevance of this framework is that it allows us to focus on the importance of each symptom within the network, thus opening venues for the exploration of (a) symptom-specific relationships with risk and protective factors, and (b) symptom-specific targets for clinical interventions.

We recently published a proof of concept analysis in which we propose to understand maternal depression as a network of interacting symptoms [26]. Applying network analyses to a community sample of Latina pregnant women, we found five strong symptom-to-symptom associations (e.g., crying—sadness), and five symptoms that were highly interconnected in the network structure. This prior work was limited in that it used a general population sample and was focused solely on cross-sectional data, rendering the temporal stability of networks unknown. This current study addresses both limitations, and we will describe (i) the network of depression symptoms in depressed women; (ii) identify the associations of maternal self-efficacy within the network of depression symptoms; and (iii) evaluate the temporal stability of the network structure.

## Methods

The Institutional Review Board of the University of North Carolina at Chapel Hill approved this study (#02–0790). This is a secondary analysis of combined data from two randomized clinical trials (RCTs) conducted from 2003–2010 in low-income communities in North Carolina and New York [28–30]. The RCTs were focused on decreasing maternal depression symptoms through an interpersonal psychotherapy/parent enhancement intervention, and the control group received either usual care or an equal attention, health education condition; both groups received parenting guidance and wrap around services as part of federal Early Head Start programming; further details about the parent RCT designs and findings can be found elsewhere [28–30]. In the parent RCTs the total sum-score of the Center for Epidemiological Studies Depression scale (CES-D) was used as a measure of change in depression severity. In the combined data, there was an effect of time on the depression symptom severity CES-D total sum-score, *F*(3, 296) = 2.903, *p* = 0.035). By the end of the study, women in both groups were still experiencing significant levels of depression symptoms as indicated by a mean severity score of 18 (SD = 12.8) on the CES-D. To account for potential effects of the intervention on the network structures, we added the intervention, as a dichotomous covariate (2 = intervention, and 1 = control), in the network analysis (i.e. we control for it statistically).

### Participants

Low-income mothers of infants or toddlers aged 6 weeks to 36 months were screened for depression symptoms using the CES-D. The sample consisted of 306 mothers whose child was enrolled in Early Head Start programs in North Carolina or New York, United States. Inclusion criteria for mothers were: 1) total sum-score of at least 16 on the CES-D, which has been extensively used as the cut-off point for high depressive symptoms [31]; 2) not receiving psychotherapy or counseling or on psychotropic medication; 3) the biological parent and primary caretaker of the index child; 4) English or Spanish speaking; 5) at least 15 years of age; and 6) at least six weeks postpartum. The last criterion was set to eliminate mothers who might have transient depression symptoms during the early postpartum period. The demographic characteristics of the dataset are reported in Table 1. There were no statistically significant differences in demographic and clinical characteristics between the intervention and control groups.

**Table 1:** Demographic and clinical characteristics of the sample.

Variable	Total Sample (n = 306)	Intervention (n = 153)	Control (n = 153)	*p*-value
Maternal age	26.1 (5.8)	26.2 (5.9)	25.9 (5.6)	0.522
Education (years)	11 (2.8)	11 (2.9)	11 (2.5)	0.118
Work status				0.859
Full- or part-time	41.3%	42.1%	40.5%	
In school	17%	17.8%	16.3%	
Neither	41.6%	40.1%	43.1%	
Race				0.479
Black/African-American	44.8%	47.7%	41.8%	
White	46.0%	45.1%	47.1%	
Mixed/Native American/Asian/Pacific Island	5.9%	5.2%	6.5%	
Unreported	3.3%	2.0%	4.6%	
Living without spouse or partner	50.5%	49.3%	51.6%	0.731
CES-D^a^ Total Sum-score	26.0 (12.5)	26.1 (12.6)	25.8 (12.2)	0.721
Child Age (months)	22.5 (13.6)	22.3 (13.8)	22.8 (13.4)	0.723
Child female gender	51.9%	54.2%	49.6%	0.492
Number of children in household ≤ 5 years old	1.8 (0.8)	1.8 (0.8)	1.9 (0.8)	0.722

^a^ CES-D = Center for Epidemiologic Studies Depression Scale.

### Measures

This analysis focused on the depression symptoms and generalized self-efficacy, which were measured over four time points at baseline (T1 –first intervention session; mean postpartum months = 22.5, SD = 13.6), 14 weeks (T2 –second/last intervention session), 22 weeks (T3 –after the last intervention session) and 26 weeks post-baseline (T4 –one month after intervention completion). The CES-D was used to measure depression symptoms [31], which is a 20-item self-report measure of depression symptom severity during the previous seven days that was designed to be used with community populations. Each symptom is scored 0 (not at all) to 3 (5–7 days), with total scores ranging from 0 to 60. Cronbach’s alpha for item consistency for the CES-D measure was ≥ .87 for each time point. Table 2 shows the mean and standard deviations of the 20 CES-D symptoms included in the analysis.

**Table 2:** Overall mean and standard deviations (SD) of the 20 CES-D symptoms included in the network analysis^1^.

Symptom	Short Codes	Mean	SD
Feeling bothered	cesd1	1.11	1.00
Appetite changes	cesd2	1.60	1.11
Feeling blue	cesd3	1.87	1.12
Lack of feeling good	cesd4	1.35	1.04
Difficulty with concentrating	cesd5	1.38	1.05
Depressed mood	cesd6	0.72	0.95
Everything was an effort	cesd7	1.28	1.15
Hopelessness	cesd8	0.81	1.02
Feeling of failure	cesd9	0.88	1.00
Fearful	cesd10	1.31	1.22
Sleep disturbances	cesd11	1.94	1.05
Lack of happiness	cesd12	0.98	1.06
Talking less	cesd13	0.69	0.99
Lonely	cesd14	1.93	0.98
People unfriendly	cesd15	1.01	0.95
Lack of enjoyment	cesd16	1.82	1.08
Crying	cesd17	0.83	1.01
Sadness	cesd18	0.98	1.12
Feeling disliked by others	cesd19	1.23	1.11
Inability to get going	cesd20	0.76	0.99

^1^ Table with mean (SD) for each time point is presented in the Table A in S1 File.

The original Generalized Self-Efficacy (GSE) scale was used to measure self-efficacy [32]. The GSE is a 10-item self-report measure designed to assess perceived self-efficacy. Each question is scored from 1 (not at all true) to 4 (exactly true). Higher scores indicate stronger patient’s belief in self-efficacy. Cronbach’s alpha for item consistence for the GSE measure was ≥ .90 for each time point. We performed a principal component analysis on the 10-item GSE scale, which suggested the item correlations in the scale can be described by one component; the first component explained 50.84%, 51.84%, 63.59% and 67.82% of the variance for T1 to T4, respectively (Fig A in S1 File). This component score was used in the analysis instead of the 10 individual GSE items because our sample of 306 participants did not have sufficient power to reliably estimate networks with 30 nodes (cf. Epskamp et al. [33] for a tutorial on and discussion of power and sample size in psychological networks).

### Data analysis

An introduction of symptom network analysis on maternal depression has been presented in Santos et al. [26]; tutorials for estimating networks are available elsewhere [33–35]. The data were analyzed via the free software environment R, and the R-code that was used for this analysis is presented as supporting material (S2 File). The data and analytical R-code are available on https://osf.io/e9r5s/, making the results fully reproducible. In this study, we performed four analyses: 1) we first estimated the network structure, which provides detailed information of the multivariate structural dependencies among variables (e.g., edge weights: the connections or lack thereof between two nodes; the type of interaction—positive or negative; and the strength of the connection between nodes); 2) to quantify the structural importance of a node in the network, we looked into the importance of individual items within the networks using centrality indices; 3) we investigated the temporal stability of the network structure across the four time points; and 4) finally, we evaluated the robustness of our findings by exploring the accuracy of edge weights and centrality indices.

#### Networks estimation

We used the R-package *qgraph* to estimate and visualize all networks [36], and followed state-of-the-art procedures to estimate regularized partial correlation networks using the Gaussian Graphical Model (GGM) for each time point [37]; a tutorial is available elsewhere [34]. For the network with the 20 CES-D nodes and the two covariates (self-efficacy and intervention), we used polychoric correlations due to the ordinal nature of the symptoms. We employed the graphical lasso (GLASSO) algorithm, which uses penalized maximum likelihood estimation, to control for spurious correlations that may arise due to multiple testing [37]. The result is a sparse network structure in which edges between nodes represent conditional dependence relations (i.e. nodes are associated after controlling for all other nodes in the network). In the resulting graph, green lines depict positive associations, and red lines negative associations. The thicker and more saturated the edge, the stronger the association between two nodes. For each of the four networks, we calculated the global strength values (i.e. the connectivity) by summing the absolute values of all edges [21].

#### Network inference

In order to gain more insight into the structural importance of items in the networks, centrality analyses were performed. Consistent with prior papers in the field, we calculated strength, betweenness and closeness centrality indices [38]. *Strength* indicates which node has the strongest overall connections and is calculated by summing the absolute edge weights that are connected to a specific node. *Betweenness* centrality reveals how often a node lies on the shortest path between all sets of two nodes in the network. *Closeness* centrality summarizes the average distance of a node to all other nodes in the network, and is calculated by the inverse of the sum of the distance from one node to all other nodes in the network.

#### Temporal network stability

Because there is currently no single test available to investigate whether more than two network structures different, we evaluated the temporal stability of the network structures over time in three different ways. First, we investigated whether the network structures of each time point differed from all other time-points by means of the Network Comparison Test with the R package *NetworkComparisonTest* [39, 40]: (A) the network structure invariance test explores differences in the structure of the network as a whole. The difference between network structures is defined as the deviation in absolute weighted sum scores of the connections [41]. This permutation-based test randomly regroups participants from the networks repeatedly and calculates the differences between the sub-networks. The resulting distribution under the null hypothesis (i.e., both networks are equal) is used to test the observed difference of the sub-networks [27, 40]. We used the dependent version of the Network Comparison Test developed for testing temporal stability; efforts to validate this test are currently under way [39]; (B) the global strength invariance test explores whether the overall level of connectivity is equal across networks. Overall connectivity is defined as the weighted absolute sum of all edges in the network [38]. The result of the network comparison tests is expressed as a *p*-value, which is set against an alpha level of 0.05 [40]. We also assessed the *similarity* of the networks by (1) correlating the adjacency matrices of the networks to assess similarities of network structures, and (2) by correlating the centrality estimates across networks. This follows the procedure of a recently published paper [42]. If the correlation among network structures equals one, networks have a perfect linear relationship, meaning that the networks have essentially the same structure; if the correlation equals zero, the networks have no detectable linear correspondence; and if the correlation equals minus one, the networks are exact opposites [43]. A similar rationale holds for the correlation among centrality coefficients.

#### Network parameter accuracy

Following a recent tutorial paper on network accuracy [33], we used the R package *bootnet* to estimate the accuracy of centrality indices by using a case-dropping subset bootstrapping approach that determines how many cases (e.g., a person in the dataset) can be removed from the network before the results become unstable, and estimated the correlation stability coefficient, which can range from 0–1: values above 0.25 imply moderate, above 0.5 strong stability. We also estimated the accuracy of edge-weights by calculating bootstrapped 95% confidence intervals (CIs) around the edge weights; smaller CIs represent more accuracy in the estimation of the edges. Finally, we tested for significant differences between all edge-weights and all centrality indices. We drew 1000 bootstraps for each routine. We note, however, that edge weights difference test and centrality difference test do not control for multiple testing.

#### Missing data

Handling of missing data for network analysis is currently an open question [44]. In the present paper, we estimated GGMs in the full dataset using pairwise complete observations. For the network comparison test, we analyzed those participants that completed the CES-D at all time points (n = 172). Since the network comparison test is a paired comparison in dependent data—and a permutation test—only participants without missing values could be included. Because the network comparison test requires considerable sample size to detect differences (i.e., it can err on the side of the H0 given small power [40]), we additionally investigated the degree to which network structures were similar by examining the correlation of the adjacency matrices for all networks using Spearman correlation coefficients.

## Results

### Networks structure

**Fig 1:**
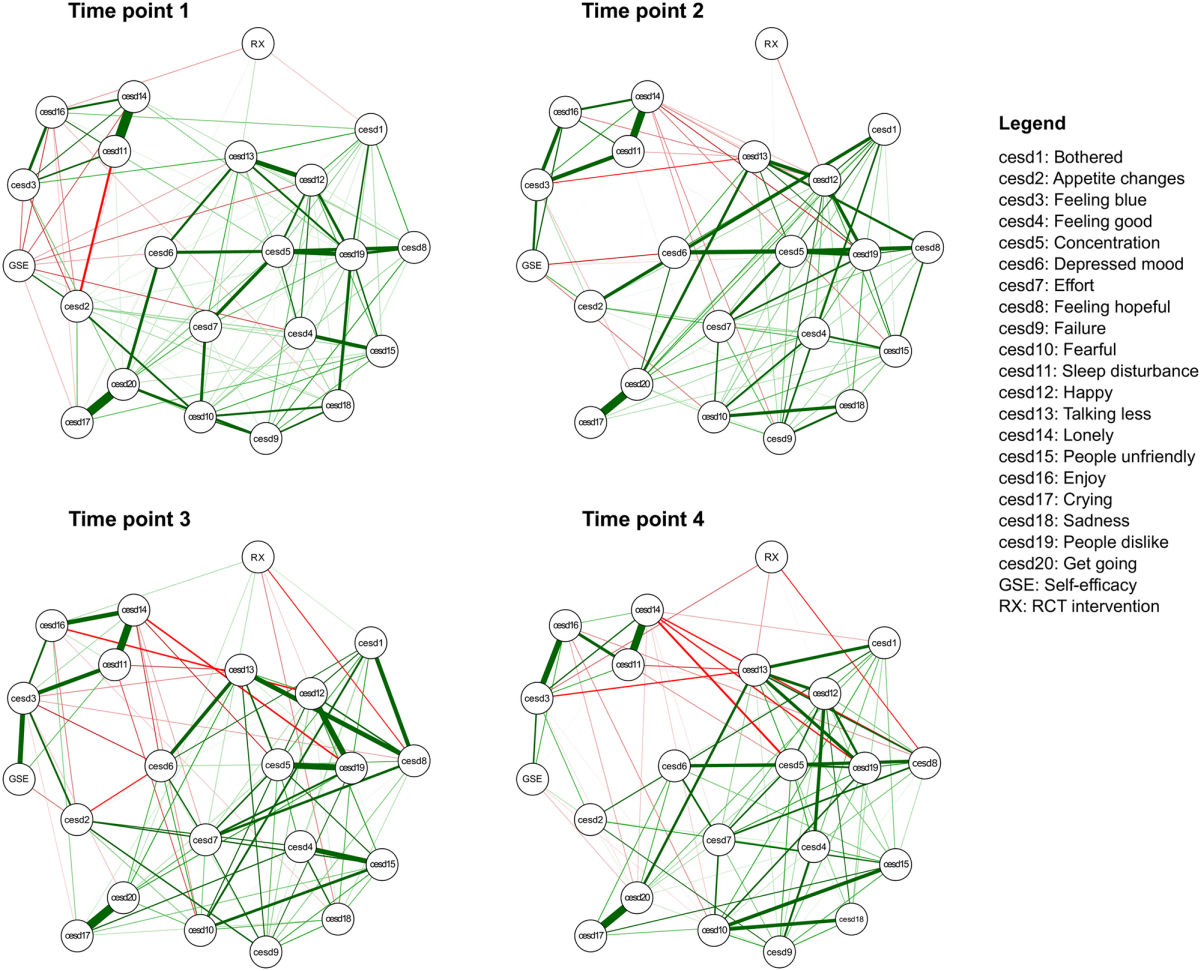
Network structure of the 20 CES-D symptoms, self-efficacy (GSE) and intervention (RX).

The estimated networks of 20 CES-D symptoms, self-efficacy (GSE) and intervention (RX) are presented in Fig 1. Overall, self-efficacy (GSE node) was weakly and negatively connected with depression symptoms. GSE and appetite changes (cesd2) had a weak and positive association that decreased in strength from T1 (0.12) to T4 (0.03). Most importantly, a positive relationship developed between GSE and feeling blue (cesd3; with the following regularized partial correlations: T1 = -0.06, T2 = 0.17, T3 = 0.29, T4 = 0.13), and this was supported by our network accuracy analysis as shown in Figs B and C in S1 File.

There were two particularly strong and consistent positive relationships among depression symptoms in the networks: lonely (cesd14)—sleep disturbance (cesd11; T1 = 0.51, T2 = 0.40, T3 = 0.40, T4 = 0.41) and inability to get going (cesd20)—crying (cesd17; T1 = 0.46, T2 = 0.44, T3 = 0.46, T4 = 0.41). In addition, concentration difficulty (cesd5)—disliked (cesd19; T1 = 0.25, T2 = 0.25, T3 = 0.34, T4 = 0.14) showed moderate and consistently relationship from T1 to T3. The intervention (RX node) was weakly connected to depression symptoms without a clear pattern over the four time points. These findings are supported by accuracy analysis (Fig B in S1 File).

### Network inference

**Fig 2:**
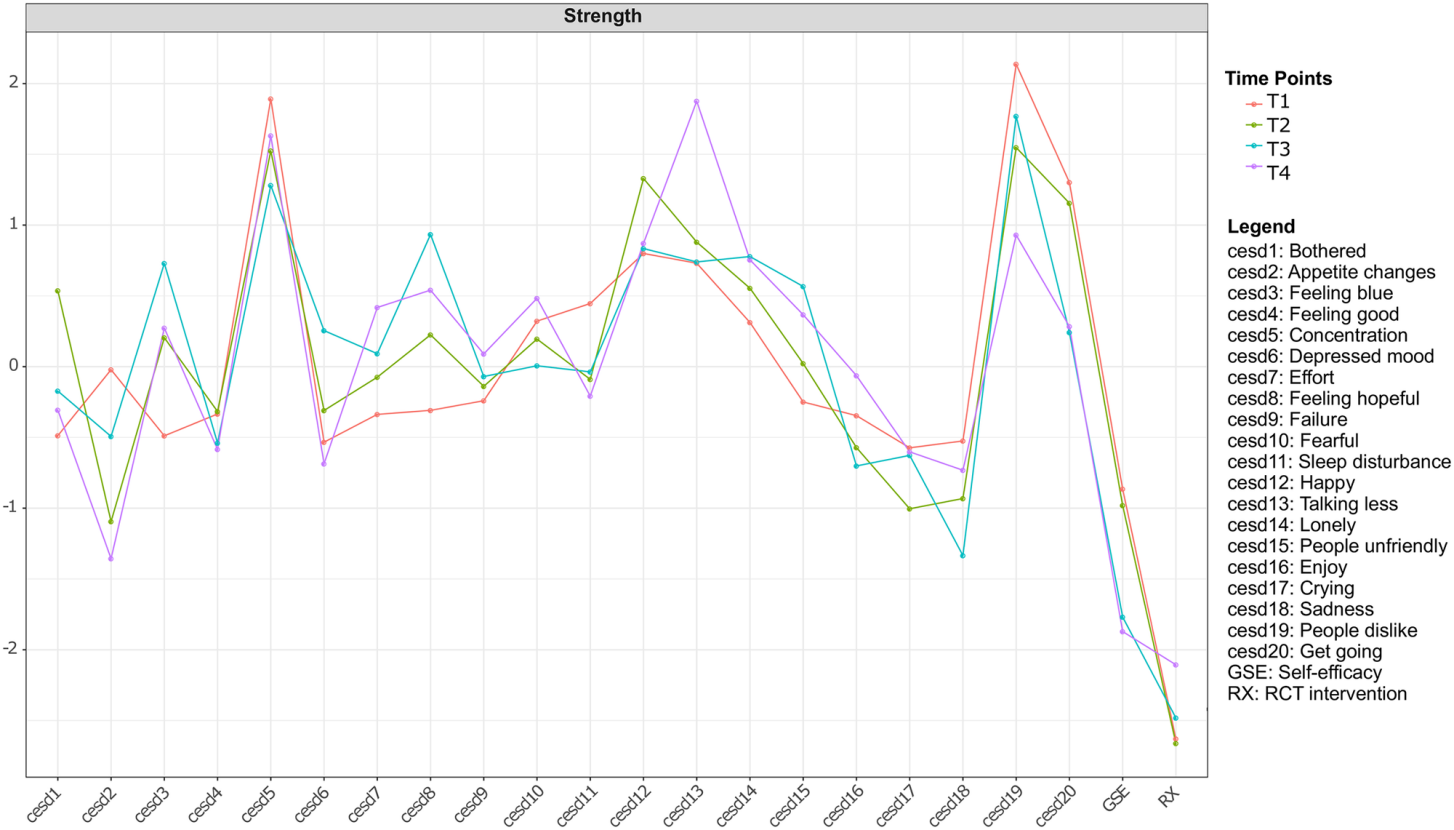
Centrality analysis of the 20 CES-D symptoms, self-efficacy (GSE) and intervention (RX).

From T1 to T4, the symptoms with highest standardized strength centrality were feeling disliked (cesd19; T1 = 1.45, T2 = 1.27, T3 = 1.34, T4 = 1.12) and concentration difficulty (cesd5; T1 = 1.38, T2 = 1.26, T3 = 1.21, T4 = 1.33) (Fig 2); centrality significance tests indicated that these symptoms had significantly higher strength than most other nodes at T1 and T2 (Figs D and E in S1 File). Inability to get going (cesd20), lack of happiness (cesd12), feeling that everything was an effort (cesd7) and talking less than usual (cesd13) were also among the symptoms with highest strength centrality from T1 to T4, respectively. Self-efficacy (GSE node) and the intervention (RX node) had very low centrality indices within the networks over time. The correlation stability coefficient for strength centrality for the four networks was 0.67, 0.60, 0.44 and 0.28 for networks 1 through 4, respectively (Fig D in S1 File); thus, T1 and T2 exceeded the recommended threshold for stable estimation of 0.5, and T3 and T4 stayed above the minimum threshold of 0.25 [33].

Betweenness and closeness did not result in stable parameters estimates (see Fig D in S1 File); we therefore chose not to interpret these centrality indices in the remainder of the manuscript. This result is consistent with prior papers: strength has always been the most precisely estimated centrality metric in psychopathology networks, and betweenness and closeness only reach the threshold for reliable estimation in large or very large samples [33, 43]. As described in the methods section, the estimation of betweenness and closeness relies on the concept of shortest path length, whereas strength centrality is simply the sum of all absolute edge values. When resampling the networks for the estimation of centrality stability, small changes in the network structure can thus lead to changes in the shortest path between two nodes, making the estimation unreliable (there are even situations where centrality indices based on the shortest path are highly unstable even in very large datasets; one example case is explained in appendix C of Borsboom et al. [43]). Small changes in the network structure when resampling, however, will only lead to small changes in strength centrality, making it a generally more robust metric.

### Temporal network stability

To evaluate the temporal stability of the networks over time (i.e., network structures), we performed three sets of analyses to look at (1) differences in network structure, (2) correlation of adjacency matrices, and (3) correlation of strength centrality (Table 3). In case the networks do not differ from each other in structure, and in case their structures and centrality coefficients are highly correlated across time, we would conclude that networks show high temporal stability. Our findings can be summarized as follows: (1) The network comparison test showed that there was a statistically significant difference in the network structure invariance test between T1 vs T3 and T2 vs T3 (i.e. the network structure did not remain exactly the same across all time points). In terms of global strength invariance test, no statistically significant difference was identified (i.e. the connectivity or density of the network remained the same across time); (2) The adjacency matrices were moderately to highly correlated, with correlations varying from 0.49 (T1 –T3) to 0.68 (T2 –T4); and the strength centrality estimates from T1 to T4 were strongly correlated (r ≥ 0.76), indicating that strength centrality was fairly stable over time. In summary, these results indicate that network structure and strength centrality show at least moderate temporal stability.

**Table 3:** Results of the network comparison test based on global strength and network invariance.

Networks		Network Invariance	Global Strength	Adjacency Matrices/Structure	
Time point	Time point	*p*-value	*p*-value	*Spearman correlation*	*p-value*
T1	T2	1	1	0.54	0.001
T1	T3	0.008	0.962	0.49	0.001
T1	T4	0.394	0.865	0.50	0.001
T2	T3	0.007	0.946	0.66	0.001
T2	T4	0.385	0.865	0.68	0.001
T3	T4	1	1	0.60	0.001

## Discussion

This is the first study to explore the multivariate structural dependencies among depression symptoms and self-efficacy in a sample of low-income mothers, and to explore the temporal stability of network structures over four time points. We found that self-efficacy and the intervention had weak relationships with the depression symptoms. For self-efficacy, most of the relationships with symptoms were negative, except the relationship with feeling blue. For intervention, no specific pattern in the relationships was identified. In terms of network structure, the strongest relationships (i.e., edge weights) were among lonely—sleep disturbance, inability to get going—crying, and concentration difficulty—feeling disliked. In terms of symptom centrality (i.e., strength centrality), feeling disliked and concentration difficulty were the symptoms with consistently high strength centrality across time. All of these findings were supported by accuracy analysis. Lastly, we found that the network structures over time were moderately stable.

### Depression symptoms and self-efficacy relationships

As expected, we found that most of the relationships between self-efficacy and depression symptoms were negative, supporting prior findings that self-efficacy is negatively related to maternal depression [8, 9]. However, due to the ability of network analysis to map out all the multivariate relationships, we additionally found that self-efficacy is *differentially* related to depression symptoms. For example, self-efficacy and appetite changes had a weak and positive association. We also observed that self-efficacy and feeling blue had a positive association. This association became stronger (i.e., edge weight) from T1 to T3. Our hypothesis is that greater self-efficacy might be related to greater capacity or greater comfort to report feeling blue. To the best of our knowledge, however, we are the first to report this specific relationship, and thus this warrant further investigation and replications in larger samples are needed. We also encourage qualitative studies to explore the meaning and contextual factors of this potential relationship between self-efficacy and feeling blue in mothers. Despite the novelty of our findings, they do fit into a growing number of publications showing that depression symptoms have differential relations to risk or protective factors, biomarkers, level of impairment and treatment response [17, 20, 26, 45, 46], which warrants the analysis of individual symptoms and their relationships instead of total sum-scores and diagnoses alone.

### Depression symptoms and intervention relationships

We included an intervention node (RX) to control for intervention effect in this analysis. We found that the intervention had weak centrality in the network, meaning that it did not strongly relate to most depression symptoms in networks, which is consistent with the findings of the combined sample of the two RCTs showing reduction of depression severity with a T4 mean still above the CES-D threshold. Discussion on the performance of this intervention on specific depression symptoms is beyond the scope of our paper; the two RCTs were intended to reduce depression severity as sum-scores and not targeted at specific depression symptoms. In addition, we did not have sufficient data to parse out in which way interventions impacted on specific symptoms, which would require time-series data [46]. We recommend the following steps for future knowledge development: First, we need more intensive data collected via ecological momentary assessment (also known as experience sampling method)—with multiple measures per day—analyzed with statistical time-series models; this would allow us to determine the direction of the effects among symptoms, along with the consistency of parameters across people [47]. In this sense, our study is the first step to generate hypotheses that can guide ecological momentary assessment studies. For example, we hypothesize that symptoms (e.g., feeling disliked, isolation) that relate to interpersonal interactions are good candidates for symptom-tailored intervention in maternal depression.

### Structural importance of symptoms in the network

Our analysis of the network structure showed that the consistently strongest edges were lonely—sleep disturbance, inability to get going—crying, and concentration difficulty—feeling disliked. In terms of symptom centrality, we found that feeling disliked and concentration difficulty were the symptoms with consistently high strength centrality across time. These findings differ from the only other available network study of maternal depression [26]. Santos et al. [26] identified lack of happiness—lack of enjoyment and feeling like people were unfriendly—feeling disliked as the strongest edges. The most central symptoms identified were depressed mood, sadness, loneliness and feeling blue and lack of happiness. We hypothesize that differences across the studies come from two potential sources. First, the network structures might differ across studied populations. In the current study, we investigated networks in a sample of ethnically diverse mothers with the indexed children having a mean age of 22 months. The prior study, on the other hand, had recruited a sample of pregnant Latina women. Second, the studies differed in average levels of depression severity, with the present study having a higher level. We utilized four time points to look into our results, and found consistent findings over time, which makes our interpretation robust.

Among the most central symptoms in our study (i.e., feeling disliked, concentration difficulty, inability to get going, lack of happiness, feeling that everything was an effort and talking less than usual), only difficulty concentrating is included in the Diagnostic and Statistical Manual of Mental Disorders, DSM-5 [48]. Other studies have found concentration impairment as an important symptom of maternal depression [49–51]. Further, concentration difficulty has been found to be one of the most impairing symptoms in terms of social and psychological functioning [19]. In a study comparing depression symptom features between non-postpartum and postpartum women, the findings suggested that impaired concentration/decision-making was one of the most prominent somatic symptoms in postpartum women [52]. Feeling disliked by others, one of the two most central symptoms in our findings, is not one commonly understood to be a depression symptom. Yet, Santos et al [26] identified isolation as one of the central symptoms in mothers with depressive symptoms. It could be that symptoms related to inter-personal relationships or perception play an important role in the well-being of mothers. This finding also suggests a broader understanding of maternal depression symptoms that goes beyond the core DSM-5 symptoms of depression, and is consistent with many prior studies that have highlighted the role of non-DSM depression symptoms in depressed samples [53, 54].

Considering the larger context of motherhood in which maternal depression takes place, it is not difficult to come up with ideas on how concentration issues, feeling disliked and isolated could play an important role as depression symptoms. Concentration issues can limit the ability of the mother to perform maternal tasks and decision making, thus leading to poor maternal-infant interaction often found in depressed mothers [55, 56]. Furthermore, mothers often feel the pressure of a cultural expectation that motherhood is a time of joyfulness and happiness; a sense of being disliked by others may be related to depressed women’s sense that they do not meet this expectation, and thus can lead to isolation. Qualitative studies have found that the discrepancies between the sociocultural expectations of motherhood and the actual experiences of women, and sense of societal group membership have been repeatedly noted as a factor to the development of maternal depression [57–59], which affects the motherhood experience.

In term of symptom centrality, we want to emphasize, however, that non-central symptoms might also be highly clinically relevant. From a network perspective, central symptoms are somewhat more interesting because in case these symptoms activate other symptoms, they might provide novel treatment targets. That being said, it is entirely possible that there are symptoms unconnected to others that are very debilitating and that cause suffering, so we do not want to broadly adequate central symptoms with clinically important symptoms. As discussed in Santos et al. [26], it is feasible that a peripheral symptom, i.e., largely unconnected in the network, could be related to impairment in daily life. It is currently unknown whether intervening on peripheral symptoms is likely to have a stronger positive impact on the whole network than intervening on a highly central symptom; if one could successful “turn off” a highly central symptom it might have a strong positive effect, but at the same time it seems unlikely to achieve such a feat given the strong interconnectedness (i.e. it would like “turn on” again due to its many connections). In summary, there is no evidence currently available to support that symptoms with low centrality are not important. Given the novelty of our network findings we recommend precaution in this translational approach until more studies can identify and confirm important central symptoms that are replicable to justify targeting them in clinical interventions. Further research is needed to understand the role of symptom centrality in driving the network of depression symptoms in mothers.

Some limitations need to be taken into consideration while interpreting the results of this study. First, parameter stability of the network needs to be kept in mind when dealing with relatively limited sample sizes. This is because a large number of parameters are estimated in regularized partial correlation models [34]. Thus, despite the fact that our findings based on 306 mothers showed at least moderate stability and accuracy, results should be considered exploratory in nature. Future research should attempt to conduct network research in multiple large datasets of maternal depression symptom to evaluate the stability and replicability of the findings [42]. Second, as previously discussed in Santos et al [26] and posed as one of the core challenges of current network models by Fried & Cramer [25], if a scale contains the same conceptual item multiple times, this might lead to biased network estimates. The CES-D includes some symptoms that are phrased similarly, and may measure the same issue with different questions, such as the items: sadness, lack of happiness, depressed mood, and feeling blue. This overrepresentation can lead to strong shared variance among these items and may thus artificially increase the centrality of these symptoms. Since none of those symptoms showed high centrality in our findings, however, we do not consider this an important concern for the present analysis. Third, we used a self-report scale as main outcome, and concerns about the reliability of self-report scales have been raised previously (however, similar concerns have been raised for clinical diagnoses; the DSM-5 field trials identified major depression as one of the least reliable diagnosis in the DSM [60]). Finally, depression rating scales differ considerably in symptom content, and an evaluation of content overlap among common depression scales indicated that the CES-D features the least representative symptoms [61, 62]. It is unclear at present which rating scales captures the proper complexity and multidimensionality of depression symptoms, and follow-up studies should aim to investigate a larger number of symptoms, including loneliness, irritability, impaired concentration, overwhelming and obsessive thoughts, and feeling guilty, that are often endorsed in post-partum populations.

This study is the first to report the network of depression symptoms and general self-efficacy in depressed mothers using data across four time points. This work expands the perinatal mental health field toward a better understanding of the complex multivariate relationships underlying maternal depression symptomatology; considering the dynamic nature of maternal depression symptom networks we put the hypothesis forward that maternal depression symptoms are phenomenologically disparate, may have distinct etiology and thus be differentially responsive to a number of treatments. Expansion on this line of research can also inform symptom-specific causal pathways and lead to symptom-tailored interventions in the future.

## Supporting information

S1 File Principal component analysis of self-efficacy, estimated accuracy and stability of the estimated networks, and mean (SD) of nodes included in Fig 1. This file contains figures showing the results of the supporting analysis for the networks presented in Fig 1, and mean (SD) for each time point used in the networks.
(DOCX)

S2 File R codes. R codes of the analysis performed.
(R) Click here for additional data file.
