# Supplementary material for: Longitudinal network structure of depression symptoms and self-efficacy in low-income mothers
Source: PLoS One. 2018 Jan 23;13(1):e0191675. doi: 10.1371/journal.pone.0191675 (PMC5779701; doi:10.1371/journal.pone.0191675)
Supplement: S1 File — This file contains figures showing the results of the supporting analysis for the networks presented in Fig 1, and mean (SD) for each time point used in the networks. (DOCX) [file pone.0191675.s001.docx]

Longitudinal network structure of depression symptoms and self-efficacy in low-income mothers

Hudson P. Santos Jr.^1*^, Jolanda J. Kossakowski,^2^ Todd A. Schwartz^1,3^, Linda Beeber^1^, Eiko I. Fried,^2^

^1^ School of Nursing, University of North Carolina, Chapel Hill, NC, USA

^2^ Department of Psychological Methods, University of Amsterdam, Amsterdam, The Netherlands

^3^ Department of Biostatistics, Gillings School of Global Public Health, University of North Carolina, Chapel Hill, NC, USA

^*^ Corresponding author:

E-mail: hsantos@unc.edu (HS)

**Fig A. Principal component analysis of the general self-efficacy (GSE) scale.**

To reduce the number of nodes in the network analysis, we performed a principal components analysis on the GSE scale, which suggested the item correlations in the scale can be described by one component, as seen in the Figure S1. We added the mean component score to the data frames in the network analysis.


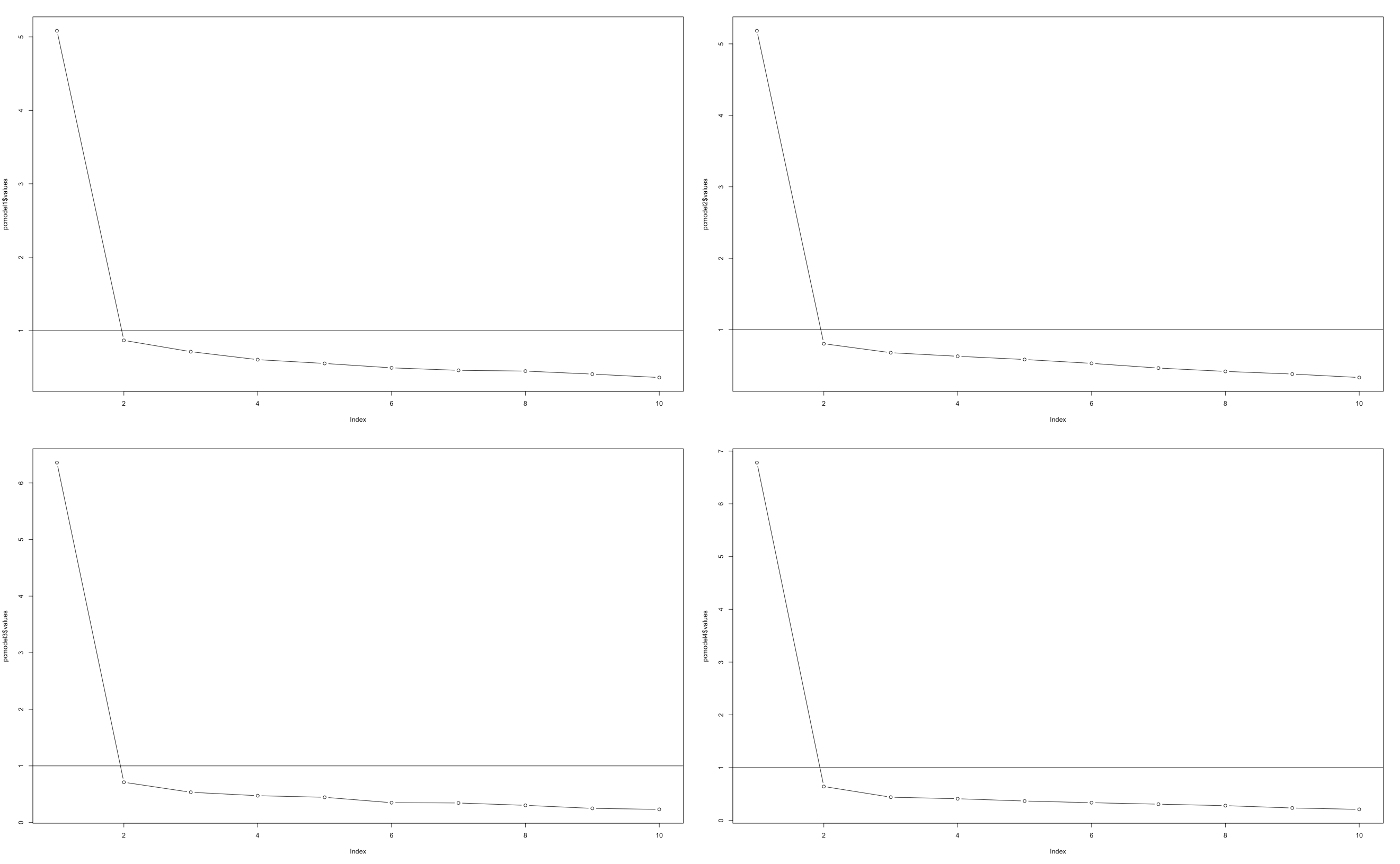


**Fig B. Accuracy of the edge-weights (solid line) and the 95% confidence intervals around these edge-eights (gray bars) of the networks from T1 to T4.**

**Fig C**. **Edge-weights difference test for the network estimated from time point 1 to 4.**

Bootstrapped difference tests (α = 0.05) between edge- weights that were non-zero in the estimated networks. Black boxes represent edges that *do* differ significantly from one-another, and gray boxes indicate edges that *do not* differ significantly from one-another. Colored boxes in the edge-weight plot correspond to the color of the edge in the networks.

**Fig D.** **Stability of centrality indices representing the average correlation of the centrality indices in the networks from T1 to T4.**

When the correlation after dropping a substantial number of participants is high, it means the centrality estimates in the original network can be considered stable.

**Fig E**. **Centrality difference test of network estimated from time point 1 to 4.**

Bootstrapped difference tests (α = 0.05) between node strength of the networks. Black boxes represent edges that *do* differ significantly from one-another, and gray boxes indicate nodes that *do not* differ significantly from one-another. White boxes in the plot show the value of node strength.

**Table A. Mean and Standard Deviations of the 20 CES-D Items from Time Point 1 to 4.**

| **Symptom** | **Short Codes** | **Time Point 1** | | **Time Point 2** | | **Time Point 3** | | **Time Point 4** | |
| --- | --- | --- | --- | --- | --- | --- | --- | --- | --- |
|  |  | **Mean** | **SD** | **Mean** | **SD** | **Mean** | **SD** | **Mean** | **SD** |
| Feeling bothered | cesd1 | 1.16 | 0.98 | 1.09 | 1.10 | 1.02 | 0.95 | 0.96 | 0.96 |
| Appetite changes | cesd2 | 1.58 | 1.10 | 1.57 | 1.20 | 1.52 | 1.13 | 1.53 | 1.09 |
| Feeling blue | cesd3 | 1.25 | 1.13 | 2.20 | 1.05 | 2.09 | 0.99 | 2.18 | 0.99 |
| Lack of feeling good | cesd4 | 1.41 | 1.03 | 1.27 | 1.14 | 1.26 | 0.98 | 1.23 | 1.02 |
| Difficulty with concentrating | cesd5 | 1.54 | 1.04 | 1.29 | 1.08 | 1.19 | 1.03 | 1.15 | 0.99 |
| Depressed mood | cesd6 | 0.75 | 0.95 | 0.65 | 1.01 | 0.66 | 0.93 | 0.63 | 0.86 |
| Everything was an effort | cesd7 | 1.42 | 1.15 | 1.24 | 1.19 | 1.08 | 1.06 | 1.05 | 1.13 |
| Hopelessness | cesd8 | 0.95 | 1.03 | 0.74 | 1.12 | 0.63 | 0.91 | 0.66 | 0.94 |
| Feeling of failure | cesd9 | 0.97 | 1.05 | 0.80 | 1.03 | 0.73 | 0.90 | 0.77 | 0.92 |
| Fearful | cesd10 | 1.44 | 1.19 | 1.18 | 1.26 | 1.13 | 1.17 | 1.20 | 1.22 |
| Sleep disturbances | cesd11 | 1.28 | 1.09 | 2.25 | 0.97 | 2.26 | 0.83 | 2.23 | 0.93 |
| Lack of happiness | cesd12 | 1.09 | 1.07 | 0.85 | 1.10 | 0.84 | 1.01 | 0.84 | 1.01 |
| Talking less | cesd13 | 0.85 | 1.01 | 0.54 | 1.02 | 0.57 | 0.91 | 0.55 | 0.88 |
| Lonely | cesd14 | 1.46 | 1.03 | 2.19 | 0.97 | 2.14 | 0.87 | 2.12 | 0.91 |
| People unfriendly | cesd15 | 1.07 | 0.91 | 0.93 | 1.05 | 0.89 | 0.89 | 0.92 | 0.94 |
| Lack of enjoyment | cesd16 | 1.42 | 1.08 | 2.00 | 1.13 | 1.99 | 1.02 | 2.03 | 1.03 |
| Crying | cesd17 | 0.83 | 0.99 | 0.82 | 1.03 | 0.77 | 0.96 | 0.76 | 1.03 |
| Sadness | cesd18 | 1.07 | 1.14 | 0.94 | 1.20 | 0.85 | 1.06 | 0.81 | 1.00 |
| Feeling disliked by others | cesd19 | 1.42 | 1.12 | 1.11 | 1.12 | 0.97 | 1.03 | 1.02 | 1.08 |
| Inability to get going | cesd20 | 0.84 | 1.05 | 0.68 | 1.06 | 0.68 | 0.87 | 0.63 | 0.87 |
